# CHF-PROM: validation of a patient-reported outcome measure for patients with chronic heart failure

**DOI:** 10.1186/s12955-018-0874-2

**Published:** 2018-03-20

**Authors:** Jing Tian, Jiangping Xue, Xiaojuan Hu, Qinghua Han, Yanbo Zhang

**Affiliations:** 1 Department of Cardiology, The 1st Hospital of Shanxi Medical University, 85 South Jiefang Road, Taiyuan, Shanxi Province 030001 China; 2 Department of Health Statistics, School of Public Health, Shanxi Medical University, 56 South XinJian Road, Taiyuan, Shanxi Province 030001 China; 3 Shanxi Medical University molecular imaging precision medicine Collaborative Innovation Center, Taiyuan, Shanxi Province 030001 China

**Keywords:** Chronic heart failure (CHF), Patient-reported outcome (PRO), Item selection, Item response theory (IRT), Reliability, Validity

## Abstract

**Background:**

Due to a lack of an appropriate disease-specific patient-reported outcome (PRO) instrument for chronic heart failure including its social support and treatment aspects in China, this study was performed to develop a patient-reported outcome measure (PROM) for patients with chronic heart failure and evaluate its reliability, validity, and feasibility.

**Methods:**

According to the standard PROM guidelines established by the Food and Drug Administration, an item pool was formed by reviewing a large amount of relevant literature and interviewing patients with chronic heart failure about their main symptoms. Thus, the primary scale was created after adjusting the items and language with the help of patients and experts in the field. Next, 155 patients from 8 hospitals in different districts were recruited for a pilot survey using questionnaires containing these items. The patients’ responses were analyzed using the classical test theory and item response theory to select high-quality items and determine the subdomains of the scale. This was followed by a formal investigation in the same eight hospitals. In total, 360 patients and 100 healthy subjects were included to evaluate the reliability, validity, and feasibility of the items. Through this process, the final scale was established.

**Results:**

The final scale comprised 12 subdomains with 57 items related to physical, psychological, social, and therapeutic areas. The data analysis results of the formal investigation showed that the PROM for chronic heart failure had good reliability, validity, and feasibility. Reliability was verified by Cronbach’s alpha coefficient, which was 0.913 for the total scale, 0.903 for the physical domain, 0.941 for the psychological domain, 0.827 for the social domain, and 0.839 for the therapeutic domain. The construct validity results met the relative criteria of confirmatory factor analysis. Discriminant validity was represented by score comparisons of nine subdomains. The response rate and the effective rate of return of the CHF-PROM were 98.94% and 98.92%, respectively.

**Conclusions:**

The final scale coincides with the theoretical framework and better reflects the overall quality of life of patients with chronic heart failure. This scale can be used as a valid instrument to evaluate clinical treatment and clinical trials of chronic heart failure.

**Electronic supplementary material:**

The online version of this article (10.1186/s12955-018-0874-2) contains supplementary material, which is available to authorized users.

## Background

Heart failure (HF) is a syndrome caused by a functional heart disorder. The heart is unable to meet the needs of the body at the normal pressure [1]. As a complex clinical syndrome, heart failure (HF) is the terminal phase of all systemic heart diseases by various causes. More than 26 million individuals have HF, and this number is increasing. By 2050, an estimated 20% people among those aged > 65 years will have developed HF [2]. HF has become an overwhelming threat to human health and social development. Based on the severity of disease, HF can be divided into acute HF (AHF) and chronic HF (CHF) [3].

CHF is the final stage of heart disease. It is a complex clinical syndrome characterized by dyspnea, edema, and fatigue [4]. Its treatment includes medical therapy, mechanical circulatory assistance, and cardiac transplantation [5]. Individual therapeutic strategies based on patients’ reported outcomes, which can reflect patients’ individual situations, has been proven effective for relieving the symptoms of CHF and improving patients’ quality of life (QoL). Compared with many other chronic diseases, CHF affects QoL more profoundly. QoL has become a major concern in modern medicine in recent years. However, clinical management and research have not taken CHF into consideration to a satisfactory degree [6]. Depression and social function disability have been shown to have a significant impact on QoL in patients with CHF [7]. Other factors affecting QoL include treatment compliance, satisfaction with treatment, and adverse effects of related treatments [8]. Additionally, decisions regarding therapy can change over time depending on the feelings of the patients and their families.

Patient-reported outcomes (PROs) are based on health-related quality of life (HRQoL). HRQoL reflects patients’ overall feelings regarding their disease and correspondent therapy. As a central part of PROs, HRQoL is essential and indispensable for evaluating patients’ health status [9]. PROs are not summaries provided by medical professionals but are instead patient-centered self-reports of patients’ feelings regarding their health state, functional status, and therapeutics. Thus, PROs are helpful in diagnosis and therapy and are of significant importance in clinical practice [10–14]. Widely accepted by medical professionals, PROs make use of patients’ feedback and view patient self-evaluation as an important aspect of the end-point in clinical trials. In 2006, the United States Food and Drug Administration circulated a publication entitled “Guidance for Industry: Patient-Reported Outcome Measures: Use in Medical Product Development to Support Labeling” [9], which further standardized the development and validation of PROs both clinically and academically [15–17].

Health-related quality of life instruments includes generic measures and disease-specific measures. All of these can reflect the quality of life of patients. General measurements for patients with chronic HF include the Nottingham Health Profile, Simple SF-36 Health Survey Questionnaire, and World Health Organization Quality of Life Scale–Brief Version [18]. These general measurements are not specific for CHF; therefore, they cannot specifically and completely represent the situation of patients with CHF. However, disease-specific measures quantify more clinically relevant domains than generic health status measures and are often more sensitive to clinical change. As the terminal phase of all organic heart diseases, CHF has specific clinical features and treatments; therefore, development of disease-specific measures for HF is necessary. Meanwhile, specific measurements used in the clinical setting include the Minnesota Living with Heart Failure Questionnaire (MLHFQ), Chronic Heart Failure Questionnaire, Kansas City Cardiomyopathy Questionnaire (KCCQ), and Quality of Life Index–Cardiac Version [18–21]. Among these, the MLHFQ and KCCQ are more popular than the others. The MLHFQ was the first questionnaire used in HF and has been translated and culturally adapted into at least 34 languages. It contains 21 items, most of which focus on physical and emotional domains; only one focuses on therapy [19, 20]. The Chronic Heart Failure Questionnaire evaluates fatigue, dyspnea, and emotion [20]. The KCCQ reports an overall summary score and five subdomain scores: physical limitations, symptoms, self-efficacy, social interference, and HRQoL. It focuses more on physical limitations, symptoms, and HRQoL and gives little attention to self-efficacy and social interference [18]. The Quality of Life Index–Cardiac Version was established in Europe and can be used for all types of heart disease [20].

Notably, doctors change treatment plans based on their patients’ social support and therapy status. For example, if the patient’s compliance decreases during the treatment period, the doctor can identify the specific cause by calculating the score of the related items in the scale. This may provide doctors with a relatively objective solution to improve patients’ dependence. Additionally, the score for the social support dimension of the scale can reflect the patient’s family situation and social environment. This could guide community doctors to help patients or their family members to solve corresponding problems and provide better community medical services. However, existing questionnaires rarely assess such factors [18, 20, 21].

Therefore, developing a Chinese questionnaire, specifically one that is culturally relevant to mainland China, is necessary because the management of CHF strongly depends on the different societal value systems, medical provision priorities, and economic environments in this country. We herein propose a measure based on PROs for patients with chronic HF to improve the current questionnaire for cardiovascular disease and guide clinical treatment.

## Methods

### 1. Establishment of CHF-PROM

#### 1.1 Conceptual framework construction

A conceptual framework for the CHF-PROM was constructed by considering the principles for developing PRO scales established by the Food and Drug Administration [22], previous life-quality questionnaires for patients with HF, and the relevant theories of CHF. The CHF-PROM should include four domains: the physical domain (PHD), psychological domain (PSD), social domain (SOD), and therapeutic domain (TRD).

#### 1.2 Item generation

We consulted a large number of relevant studies and related questionnaires [9, 18–22]. The patients’ major disease symptoms, psychological and social conditions, and satisfaction towards medical services or side effects of treatment were also collected. The item pool was generated according to all of this information.

#### 1.3 Formation of preliminary scale

Face-to face interviews regarding the above-mentioned items were required. Patients’ subjective opinions were taken into consideration. The item pool was applied to 10 patients with CHF in hospitals or communities (5 males, 5 females; average age, 65 years). During this process, the patients were asked to point out words they could not understand, and items were added or deleted as necessary. The items were revised by three cardiovascular disease experts, a psychologist, and a sociologist, who were invited to make suggestions regarding all four domains. Based on the patients’ and experts’ opinions, the CHF-PROM was further modified to form a preliminary scale. The scores of the items were calculated using a 5-point Likert scale.

### 1.4 Determination of the preliminary scale and formation of the final scale

#### 1.4.1 Survey sample and sample size

Patients were enrolled from eight different hospitals in Shanxi Province, China. The inclusion criteria for this study were an age of > 18 years, with the principal diagnosis of Chronic Heart Failure according to the 2013 ACC/AHA guideline on HF [2], and consent to fill out the questionnaire. We excluded patients with combined psychiatric disorders and those who were incapable of understanding or completing the questionnaire because of language barriers or intellectual disabilities. Healthy subjects were defined as people who had not been diagnosed with any diseases by physicians. Healthy subjects who matched the basic characteristics of patients with CHF were recruited from communities of Shanxi Province. Before collecting healthy subjects, the investigators contacted related departments of target communities to obtain support from community workers. At the same time, full preparations for publicity were made by creating posters to display in the communities. Documents that introduced the survey were also distributed. Healthy subjects who were willing to participate in the questionnaire survey provided written informed consent. The participants filled out the questionnaire by following the same survey process followed by patients with CHF. In cases of missing, we corrected and supplemented the data in a timely manner. In factor analysis, Nunnally [23] suggested that the number of subjects should be at least 10 times the number of study variables. Some scholars have suggested that the actual sample size should be 5 to 10 times greater than the number of observed variables to obtain accurate parameter estimates and reliable results [24].

The purpose of our study was thoroughly explained to all participants. Written informed consent was obtained from all participants. These questionnaires were made available on the first day of hospitalization. During hospitalization, the patients independently completed the questionnaires according to their own physical conditions by following the instructions provided by the investigators. For the elderly patients who were unable to complete the questionnaires, the investigators read the content of the questionnaires and/or filled in the answers according to the patients’ selections without any suggestions. Data entry and its verification are important in the process of data management in clinical studies [25]. Double data entry was adopted to control data quality using EpiData3.1 software. In total, 105 patients and 50 healthy subjects were enrolled in the pilot study. Various statistical analyses were conducted to select high-quality items and develop the preliminary scale, such as the classical test theory [e.g., discrete trend, factor analysis, correlation coefficient, Cronbach’s α if item deleted (CAID) and corrected item-total correlation (CITC)] and item response theory. A further larger-scale survey involving 365 patients with CHF and 100 healthy subjects was conducted by using the preliminary scale.

#### 1.4.2 Scale scoring

Patients responded to each item on a 5-point Likert scale to reflect how often they had experienced each issue during the past 2 weeks. An initial value ranging from 0 to 4 was assigned for each category (0 = never, 1 = occasionally, 2 = about half of the time, 3 = often, and 4 = almost every day). To ensure a consistent relationship between the responses to all items and the PROM, all responses were transformed in the following way: positively scored items were recorded as the original score plus 1, while negatively scored items were recorded as 5 minus the original score. This resulted in a score ranging from 1 to 5 for each item, with a higher score associated with a more positive PROM.

#### 1.4.3 Item reduction based on both CTT and IRT

### Discrete trend

A low discrete degree indicated that the subjects were inclined to select the same answer. In other words, the items were not useful for indicating differences. The scores generally exhibited a normal distribution; thus, the standard deviation was calculated for every item. Items with a low standard deviation (< 1.0) were deleted. Generally, a value of > 1.0 indicates that the participants may select different answers for an item [26].

### Exploratory factor analysis

Considering the small sample size, an exploratory factor analysis was performed and the solution was rotated separately in each field (physical, psychological, social, and therapeutic). We determined the number of factors according to the eigenvalue and variance contribution ratio. The eigenvalue should be > 1.0, and the maximum cumulative variance contribution rate was 70%. Items with low factor loading (< 0.4) were removed. Generally, it was considered that the measurable variable (e.g., item) was mainly affected by this potential factor (e.g., subdomain) if factor loading was ≥0.4 [27].

### CAID

The CAID and CITC were used to evaluate the internal consistency among the items. If an item had a negative effect on the internal consistency of its own dimension, Cronbach’s α coefficient increased greatly when the item was deleted. A CITC of < 0.4 indicated that an item was poorly correlated to the scale. In this circumstance, the item should be deleted [28].

### Correlation coefficient

The representativeness of an item was measured by the correlation coefficient with its own subdomain. An item with a correlative value of < 0.6 was generally considered to be poorly correlated to the corresponding subdomain [29]. Such an item was removed.

### IRT

IRT is part of modern measurement theory and was proposed to overcome the defects of CTT [30]. It is also called latent trait theory and has advantages in terms of item selection and test construction. It claims that the relationship between subjects’ abilities and their responses to an item can be described as a function. The basic task is to define this relationship. In brief, IRT can be viewed as a probabilistic method for discussing the relationship between subjects’ potential traits and their responses to items.

If we set *θ* as a subject’s ability, then *p*(*θ*) is the probability that the subject will respond to an item correctly. The functional relationship can be reflected by a curve called the item characteristic curve. We selected two important parameters on the curve: *α* reflects the discriminant degree, and *b* indicates the item difficulty. A graded response model appropriate for hierarchical and continuous data was constructed considering the 5-point Likert scale used in this study, extending a unidimensional model to a multidimensional one [31]. Five parameters were estimated in our study, namely *a*, *b*_1_, *b*_2_, *b*_3_, and *b*_4_, where *b*_1_ is the difficulty level parameter between Answers 1 and 2, and so on, and *b*_1_ < *b*_2_ < *b*_3_ < *b*_4_. Here, *a* must have a value of > 0.60, and *b* ranges from − 3 to 3. Items supported by at least three methods were retained in the final CHF-PROM.

### 2. Validation of the final scale

#### Reliability

We calculated Cronbach’s alpha coefficients for four fields and the total scale to measure the internal consistency of the CHF-PROM. Generally, a value of > 0.70 indicates that individual items provide an adequate contribution to the overall scale [32].

### Validity

#### Content validity

The patients’ opinions were typically consulted to validate the content with respect to how well the items met the empirical indexes of interest [33].

#### Construct validity

We subjected the factor structure of the scale to confirmatory factor analysis (CFA). The model was assessed with respect to the following relative goodness-of-fit statistics: root mean square error approximation (values of < 0.08 indicated adequate fit and values of < 0.05 indicated close fit of the data to the model) [34], normed fit index (values of ≥0.90), non-normed fit index (values of ≥0.90), incremental fit index (values of ≥0.90), comparative fit index (values of ≥0.90), and root mean square residual (values of < 0.09) [33]. We used LISREL 8.70 to assess the construct validity with CFA.

#### Discriminant validity

We determined the discriminant validity by comparing the mean scores for every subdomain of the CHF-PROM among the healthy people and patients with CHF. We compared the differences using a *t*-test, with the significance level set at *P* < 0.05 [35].

### Feasibility

**Fig. 1:**
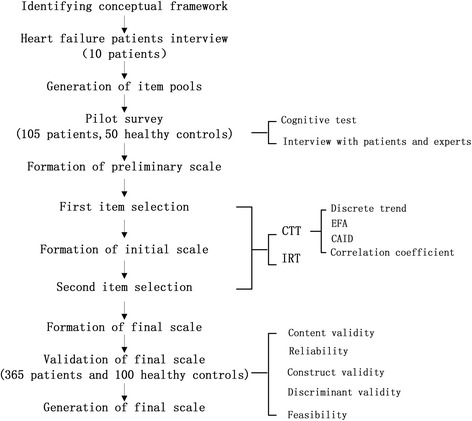
Study flow diagram

We evaluated the feasibility of the CHF-PROM by examining the response rate, completion rate, response time to completion, percentage of missing data, and score distribution. We considered response and return rates of < 85% to be inadequate and a completion time of 30 min to be acceptable. SPSS 16.0, Multilog 7.03, EpiData3.1, and LISREL 8.70 were used to conduct the data analysis. The entire study flow diagram is present in Fig. 1.

## Results

### Generation of item pool

**Fig. 2:**
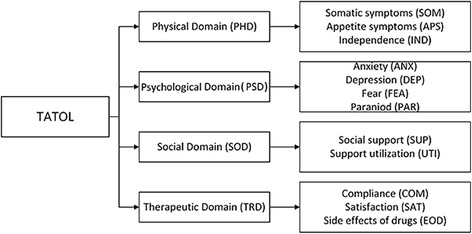
Conceptual framework of the CHF-PROM

After consulting relevant literature and interviewing patients with CHF, we established four domains as described in the Methods section: physical domain, psychological domain, social domain, and therapeutic domain. These 4 domains were then divided into 12 subdomains and a pool of 67 items (see Additional file 1). The conceptual framework of the instrument is shown in Fig. 2.

### Formation of preliminary scale

Establishment of the CHF-PROM was based on published literature and related questionnaires. Consultants were also needed to improve the validity of the questionnaire [3, 7, 8, 12–15]. According to the advice provided by patients and experts in this field, six items were removed (“PHD1. Do you feel that your limb is weak?”, “PHD15. Do you have constipation?,” “PSD13. Do you often check things over and over again?,” “PSD14. Do you often wash your hands or count over and over again?,” “PSD22. Do you feel that people do not judge your achievements properly?,” and “TRD6. Did you think the examinations are necessary?”), three items were added (“Do you feel that your illness is a burden to your family?,” “Do you know the side effects of the drugs?,” and “Are you worried about the side effects of the drugs?”), and one item was divided into two items (“PSD4. Do you feel less concentrated and forget things easily?”). As a result, we generated 65 items for the CHF-PROM.

### Item selection

#### Participant characteristics

The screening phase involved 105 patients and 50 healthy subjects. The patients with CHF had an average age of 69.16 ± 11.24 years. The normal subjects had an average age of 56.96 ± 14.96 years. The basic characteristics of the patients with CHF and healthy subjects are shown in Table 1. The demographic data were compared using the chi-square test for categorical variables.

**Table 1:** Demographic characteristics of the participants in the item-selection phase

Variables	Group	Case	Control	*x* ^2^	*P*
Sex	Female	45(42.9)	24(48.0)	1.059	0.589
	Male	60(57.1)	26(52.0)		
Marital status	Single	4(3.8)	3(6.0)	1.570	0.814
	Married	78(74.3)	39(78.0)		
	Separated	3(2.9)	1(2.0)		
	Divorced	3(2.9)	2(4.0)		
	Widowed	17(16.2)	5(10.0)		
Education level	No formal education	13(12.4)	1(2.0)	6.125	0.190
	Primary school	16(15.2)	10(20.0)		
	Junior high school	31(29.5)	20(40.0)		
	Senior high school	25(23.8)	12(24.0)		
	College or higher	20(19.0)	7(14.0)		
Occupation	Peasant	8(7.5)	3(6.0)	9.795	0.081
	Worker	5(4.7)	7(14.0)		
	Management	58(54.7)	16(32.0)		
	Researchers	18(17.0)	13(26.0)		
	Business	16(15.1)	10(20.0)		
	Other	1(0.9)	1(2.0)		
Monthly income	<$150	20(19.0)	8(16.0)	2.774	0.428
	$150–$300	57(54.3)	24(48.0)		
	$300–$450	14(13.3)	12(24.0)		
	>$450	14(13.3)	6(12.0)		
Smoking per day	Never	5(4.8)	0(0.0)	5.516	0.238
	< 10 cigarettes	63(60.0)	27(54.0)		
	10–20 cigarettes	19(18.1)	15(30.0)		
	20–40 cigarettes	13(12.4)	7(14.0)		
	> 40 cigarettes	5(4.8)	1(2.0)		

Data are presented as n (%) participants

#### First item-selection phase

Five statistical methods within the CTT and IRT were used to select the items. Items PHD3, PHD7, PSD12, and SOD9 were deleted according to the above-mentioned criteria. As a result, the initial scale contained 61 items, 10 subdomains, and 4 domains.

#### Second item-selection phase

**Fig. 3:**
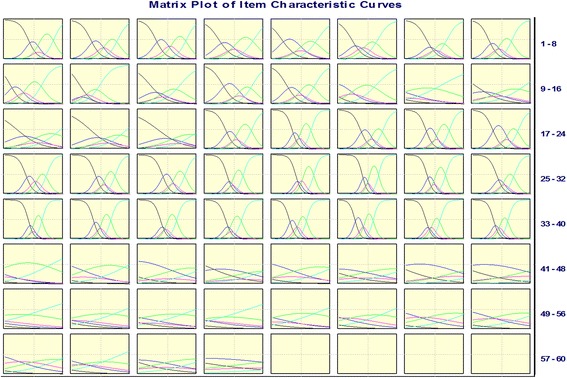
Item characteristic curve matrix. 1: Black, 2: Blue, 3: Magenta, 4: Green, 5:Cyan

As shown in Table 2, PHD9, PHD10, PHD14, PSD2, PSD18, PSD19, PSD20, PSD21, SOD1, and TRE1 were deleted according to the discrete trend ( s < 0.96). PHD4, PHD5, PHD8, PHD9, PHD10, PHD13, PHD14, and PHD15 were removed according to the factor analysis. PHD9, PHD10, TRE11, and TRE12 were deleted because the correlation coefficient was < 0.6. We also deleted PHD16 and SOD6 based on the CAID method. SOD6, SOD8, TRE1, TRE2, TRE3, TRE4, TRE11, and TRE12 were eliminated according to IRT. Figure 3 shows the item characteristic curve matrix of each item. Items proposed by at least three methods were retained. The final scale contained 57 items, 12 subdomains, and 4 domains (see Additional file 2). The final construction frame is shown in Table 3.

**Table 2:** Summary of the second item-selection phase using CTT and IRT

Item	SD	Factor analysis	Correlation coefficient	CICT	IRT					Outcome
					*a*	*b*_1_	*b*_2_	*b*_3_	*b*_4_	
PHD1	1.207	0.509	0.812	0.871	2.19	−0.60	0.27	0.60	2.00	√
PHD2	1.147	0.540	0.831	0.868	1.98	−1.05	−0.03	0.58	1.72	√
PHD3	1.051	0.618	0.755	0.876	1.98	−1.24	−0.13	0.56	1.90	√
PHD4	1.064	**0.222**	0.752	0.876	1.49	−1.62	− 0.41	0.26	1.96	√
PHD5	1.109	**0.187**	0.731	0.878	1.41	−2.00	−0.40	0.22	1.62	√
PHD6	0.997	0.763	0.680	0.882	1.66	−1.71	−0.87	− 0.15	0.97	√
PHD7	1.234	0.690	0.728	0.880	1.83	−0.89	−0.20	0.29	1.21	√
PHD8	0.978	**0.385**	0.659	0.883	2.10	−1.39	−0.58	−0.09	1.05	√
PHD9	**0.770**	**0.195**	**0.558**	0.889	1.71	−2.51	−1.41	−0.75	0.79	
PHD10	**0.745**	**0.263**	**0.543**	0.889	1.55	−2.20	−1.72	−1.10	−0.09	
PHD11	1.189	0.588	0.636	0.693	1.14	−1.85	−0.76	−0.13	1.29	√
PHD12	1.217	0.421	0.688	0.664	1.65	−1.23	0.19	0.71	1.84	√
PHD13	1.082	**0.257**	0.777	0.584	1.68	−1.55	−0.45	0.11	1.40	√
PHD14	**0.839**	**0.055**	0.657	0.645	1.05	−2.33	−1.16	0.17	1.87	
PHD15	0.961	**0.146**	0.635	0.661	0.58	**−6.29**	−2.96	−2.28	0.77	√
PHD16	1.231	0.588	0.825	**0.916**	0.68	− 2.94	−1.05	0.16	2.55	√
PHD17	1.315	0.421	0.914	0.870	0.83	−1.75	−0.11	0.69	2.70	√
PHD18	1.387	**0.257**	0.936	0.857	0.89	−1.10	0.25	0.95	2.63	√
PHD19	1.403	**0.055**	0.881	0.894	0.76	−1.02	0.46	1.20	**3.29**	√
PSD1	1.101	0.588	0.701	0.874	2.21	−0.95	−0.06	0.38	1.74	√
PSD2	**0.937**	0.421	0.689	0.874	3.01	−1.00	−0.41	− 0.05	0.99	√
PSD3	1.183	**0.257**	0.716	0.873	2.38	−0.81	0.11	0.55	1.52	√
PSD4	1.074	**0.055**	0.810	0.860	2.59	−0.99	−0.07	0.32	1.48	√
PSD5	1.175	**0.146**	0.784	0.864	2.27	−0.98	0.24	0.67	1.69	√
PSD6	1.116	0.588	0.769	0.865	2.56	−0.89	−0.13	0.41	1.29	√
PSD7	1.205	0.421	0.719	0.873	2.64	−0.61	0.12	0.50	1.49	√
PSD8	1.208	**0.257**	0.760	0.867	2.55	−0.59	0.30	0.72	1.78	√
PSD9	0.998	0.709	0.778	0.886	2.95	−0.83	−0.38	−0.08	0.89	√
PSD10	1.050	0.703	0.834	0.875	3.25	−0.63	−0.22	0.20	1.28	√
PSD11	1.016	0.819	0.886	0.863	3.79	−0.65	−0.18	0.11	1.06	√
PSD12	0.973	0.712	0.804	0.880	3.20	−0.87	−0.27	0.03	1.08	√
PSD13	1.236	0.681	0.778	0.894	2.53	−0.58	0.01	0.39	1.27	√
PSD14	1.059	0.757	0.821	0.878	3.44	−0.63	−0.22	0.01	0.82	√
PSD15	1.002	0.541	0.887	0.829	3.18	−0.83	−0.27	0.09	1.03	√
PSD16	0.977	0.599	0.905	0.788	3.05	−0.80	−0.39	−0.06	0.92	√
PSD17	0.915	0.602	0.878	0.825	2.78	−0.98	−0.52	−0.26	0.71	√
PSD18	**0.768**	0.841	0.861	0.848	3.22	−1.31	−0.70	−0.28	0.53	√
PSD19	**0.702**	0.814	0.883	0.837	3.48	−1.43	−0.76	−0.37	0.51	√
PSD20	**0.860**	0.725	0.872	0.847	3.19	−1.00	−0.63	−0.24	0.51	√
PSD21	**0.920**	0.673	0.850	0.873	3.27	−0.91	−0.47	−0.11	0.63	√
SOD1	**0.816**	0.658	0.686	0.820	0.50	**−5.37**	−2.93	−1.73	1.74	√
SOD2	1.003	0.758	0.783	0.791	0.51	**−7.36**	−2.60	−1.20	2.94	√
SOD3	1.163	0.803	0.844	0.768	0.49	**−6.00**	−0.51	0.68	**4.30**	√
SOD4	1.346	0.694	0.777	0.820	0.46	**−3.09**	0.34	1.49	**4.40**	√
SOD5	1.074	0.739	0.798	0.787	0.48	**−6.85**	−1.80	−0.19	**3.76**	√
SOD6	1.226	0.578	0.746	**0.859**	0.40	−2.79	−0.31	0.52	**3.10**	√
SOD7	1.131	0.806	0.864	0.569	0.41	**−4.39**	1.03	2.87	**7.13**	√
SOD8	1.107	0.823	0.861	0.571	**0.35**	−2.05	0.68	1.61	2.83	√
TRE1	**0.935**	0.789	0.856	0.704	**0.32**	−2.88	−1.09	−0.08	1.00	√
TRE2	1.035	0.722	0.846	0.744	0.41	**−4.90**	−2.93	−1.72	2.43	√
TRE3	1.128	0.760	0.855	0.764	0.40	**−6.56**	**−3.05**	−1.48	2.66	√
TRE4	1.030	0.734	0.821	0.917	**0.32**	**−10.72**	−2.77	− 1.84	1.28	√
TRE5	1.021	0.799	0.868	0.911	**0.39**	**−8.22**	−2.96	−2.06	2.42	√
TRE6	1.000	0.847	0.890	0.908	0.42	**−8.06**	**−3.88**	−1.11	2.61	√
TRE7	0.999	0.847	0.858	0.912	0.41	**−8.53**	−2.85	−0.03	**4.25**	√
TRE8	1.042	0.810	0.770	0.924	0.41	**−8.17**	−2.54	0.12	**4.49**	√
TRE9	1.016	0.799	0.798	0.920	0.44	**−8.33**	−2.89	0.14	**3.80**	√
TRE10	1.055	0.824	0.831	0.916	0.40	**−8.61**	**−3.80**	−1.30	2.17	√
TRE11	1.242	0.817	**0.432**	0.793	0.40	**−5.04**	−1.05	0.77	2.95	√
TRE12	1.218	0.830	**0.394**	0.793	**0.33**	−1.66	0.05	1.53	2.65	√

Note: The bold values do not meet the corresponding criteria of the methods of CTT and IRT

**Table 3:** Structure of the 57 items in the final scale

Domain	Subdomain	Item
Physical domain	somatic symptoms	PHD1−, PHD2−, PHD3−, PHD4−, PHD5−, PHD6−, PHD7−, PHD8−
	appetite symptoms	PHD9−, PHD10−, PHD11−, PHD12−
	independence	PHD13, PHD14, PHD15, PHD16
Psychological domain	anxiety	PSD1−, PSD2−, PSD3−, PSD4−, PSD5−, PSD6−, PSD7−, PSD8−
	depression	PSD9−, PSD10−, PSD11−, PSD12−, PSD13−, PSD14−
	fear	PSD15−, PSD16−, PSD17−
	paranoia	PSD18−, PSD19−, PSD20−, PSD21−
Social domain	social support	SOD1, SOD2, SOD3, SOD4, SOD5
	support utilization	SOD6, SOD7, SOD8
Therapeutic domain	compliance	TRE1, TRE2
	satisfaction	TRE3, TRE4, TRE5, TRE6, TRE7, TRE8, TRE9, TRE10
	side effects of drugs	TRE11, TRE12-

The negative sign represents the reverse score of the item

### Validation of the scale

The scale was validated in large-scale sample. The sample size was determined based on Nunnally’s rule. The sample size was only slightly below the target sample size. Patients were enrolled from different departments of eight different hospitals in Shanxi Province, China. Some patients were not willing to participate in the questionnaire because of their physical condition at that time, fear of disclosing their privacy, and other factors. In these target hospitals, several departments of cardiology were participating in investigations using other psychological questionnaires and were therefore unwilling to take part in the survey. Bias many be introduced into the study results if inpatients with CHF participate in two questionnaires simultaneously. So, 470 questionnaires were sent out and 467 were collected (98.50%) totally. There were 460 valid questionnaires (patients with CHF, 360; healthy people, 100). The patients with CHF had an average age of 69.87 ± 10.60 years, and the healthy subjects had an average age of 57.06 ± 14.67 years. The participants’ baseline data are shown in Table 4. The demographic data were compared using the chi-square test for categorical variables.

**Table 4:** Baseline data of the participants in the formal survey

Variables	Group	Case	Control	*x* ^2^	*P*
Sex	Female	154 (42.8)	54(54.0)	3.979	0.054
	Male	206(57.2)	46(46.0)		
Marital status	Single	33 (8.1)	6 (12.0)	8.694	0.069
	Married	269(66.3)	23 (46.0)		
	Separated	22(5.4)	5 (10.0)		
	Divorced	24 (5.9)	6 (12.0)		
	Widowed	58 (14.3)	10 (20.0)		
Education level	No formal education	55 (15.6)	10 (10.0)	3.909	0.418
	Primary school	80 (22.7)	26 (26.0)		
	Junior high school	81 (23.0)	29 (29.0)		
	Senior high school	77 (21.9)	22 (22.0)		
	College or higher	59 (16.8)	13 (13.0)		
Occupation	Peasant	43(12.2)	11 (11.0)	4.462	0.461
	Worker	159 (45.2)	37 (37.0)		
	Management	61 (17.3)	18 (18.0)		
	Researcher	33 (9.4)	13 (13.0)		
	Business	20 (5.7)	5 (5.0)		
	Other	36 (10.2)	16 (16.0)		
Monthly income	<$150	77 (21.4)	23 (23.0)	5.314	0.150
	$150–$300	156 (43.3)	34 (34.0)		
	$300–$450	86 (23.9)	22 (22.0)		
	>$450	41 (11.4)	21 (21.0)		
Smoking per day	Never	178 (49.4)	49 (49.0)	1.746	0.782
	< 10 cigarettes	79 (21.9)	24 (24.0)		
	10–20 cigarettes	63 (17.5)	20 (20.0)		
	20–40 cigarettes	30 (8.3)	5 (5.0)		
	> 40 cigarettes	10 (2.8)	2 (2.0)		

#### Reliability

Cronbach’s alpha coefficients for the four domains and overall scale are shown in Table 5. In general, this questionnaire showed great reliability.

**Table 5:** Reliability of the four domains and overall scale

Domain	Cronbach’s alpha coefficients
Physical domain	0.903
Psychological domain	0.941
Social domain	0.827
Therapeutic domain	0.839
Total	0.913

#### Construct validity

The results of the CFA were as follows: physical domain measurement model: 16 items corresponding to 3 latent variables; PSD measurement model: 21 items corresponding to 4 latent variables; SOD measurement model: 8 items corresponding to 2 latent variables; and TRD measurement model: 12 items corresponding 3 latent variables. Table 6 shows the goodness-of-fit for the CFA. The results showed that the model correlated well with the reference standard. The parameter estimation results of the CFA are presented in Table 7.

**Table 6:** Goodness-of-fit statistic in the four domains and total scale of the CHF-PROM

Domain	RMSEA	RMR	NFI	NNFI	CFI	IFI
Physical domain	0.09	0.08	0.88	0.87	0.89	0.89
Psychological domain	0.08	0.06	0.95	0.95	0.96	0.96
Social domain	0.09	0.08	0.88	0.83	0.89	0.89
Therapeutic domain	0.10	0.05	0.92	0.91	0.93	0.93
Total	0.08	0.07	0.90	0.93	0.93	0.93

CFI = comparative fit index, IFI = incremental fit index, NFI = normed fit index, NNFI = non-normed fit index, RMR = root mean square residual, RMSEA = root mean square error of approximation

**Table 7:** Results of the CFA

Dimension	Item	Nonstandard Factor Loading	SE	Standard Factor Loading	*t*
SOM	PHD1	0.74	0.04	0.89	21.05
	PHD2	0.96	0.04	0.9	21.48
	PHD3	0.71	0.04	0.79	17.59
	PHD4	0.62	0.04	0.75	16.4
	PHD5	0.76	0.05	0.74	16.07
	PHD6	0.73	0.05	0.69	14.64
	PHD7	0.87	0.06	0.7	14.95
	PHD8	0.6	0.04	0.65	13.47
APS	PHD11	0.58	0.06	0.50	9.27
	PHD12	0.74	0.06	0.64	12.44
	PHD13	0.80	0.05	0.84	17.39
	PHD15	0.53	0.05	0.53	9.95
IND	PHD16	1.20	0.07	0.80	17.90
	PHD17	1.27	0.06	0.92	22.52
	PHD18	1.49	0.06	0.97	24.68
	PHD19	1.34	0.07	0.87	20.38
ANX	PSD1	0.57	0.04	0.69	14.59
	PSD2	0.62	0.04	0.74	15.97
	PSD3	0.75	0.05	0.71	15.12
	PSD4	0.70	0.04	0.83	18.94
	PSD5	0.85	0.05	0.78	17.16
	PSD6	0.88	0.05	0.79	17.4
	PSD7	0.75	0.05	0.73	15.68
	PSD8	0.82	0.05	0.77	16.86
DEP	PSD9	0.74	0.04	0.82	18.57
	PSD10	0.71	0.03	0.87	20.43
	PSD11	0.76	0.03	0.92	22.72
	PSD12	0.67	0.04	0.83	19.15
	PSD13	0.84	0.05	0.76	16.68
	PSD14	0.82	0.04	0.83	19.02
FEA	PSD15	0.76	0.04	0.87	20.24
	PSD16	0.77	0.04	0.90	21.4
	PSD17	0.77	0.04	0.89	20.9
PAR	PSD18	0.88	0.04	0.92	22.74
	PSD19	0.78	0.03	0.94	23.58
	PSD20	0.92	0.05	0.86	20.00
	PSD21	0.87	0.05	0.83	19.19
SUP	SOD1	0.81	0.05	0.73	15.34
	SOD2	0.67	0.04	0.83	18.57
	SOD3	0.57	0.03	0.84	18.57
	SOD4	0.71	0.05	0.68	13.89
	SOD5	0.55	0.03	0.77	16.62
UTI	SOD6	0.51	0.05	0.49	9.42
	SOD7	0.69	0.04	0.90	18.72
	SOD8	0.71	0.04	0.89	18.54
COM	TRE1	1.37	0.07	0.90	20.79
	TRE2	1.20	0.06	0.84	18.65
	TRE3	0.98	0.06	0.79	17.10
SAT	TRE4	0.90	0.04	0.88	20.84
	TRE5	0.92	0.04	0.92	22.53
	TRE6	0.83	0.04	0.93	22.83
	TRE7	0.60	0.03	0.83	18.96
	TRE8	0.58	0.04	0.73	15.84
	TRE9	0.50	0.03	0.75	16.54
	TRE10	0.66	0.03	0.88	20.96
EOD	TRE11	1.01	0.08	0.92	12.13
	TRE12	0.80	0.07	0.78	11.13

#### Discriminant validity

In this survey, the scores of each subdomain in addition to the therapeutic domain and total score of the scale between the patients and healthy subjects showed significant differences (see Table 8). These differences indicated that the scale was able to distinguish people in different groups.

**Table 8:** Score comparisons between healthy subjects and patients with CHF

Subdomain	Case	Control	Cohen’s d	*t*	*P*
Somatic symptoms	29.96 ± 6.60	32.02 ± 6.60	0.31	2.242	0.025
Appetite sleep	15.49 ± 3.11	18.54 ± 5.08	0.84	6.389	0.001
Independence	12.96 ± 4.75	31.38 ± 6.10	3.63	26.617	0.001
Anxious	29.29 ± 6.69	31.38 ± 6.10	0.32	2.269	0.024
Depressed	24.13 ± 5.17	25.54 ± 5.08	0.27	7.891	0.001
Fear	12.65 ± 2.58	14.02 ± 1.36	0.58	10.830	0.001
Paranoid	17.89 ± 2.81	20.85 ± 2.62	1.07	41.872	0.001
Social support	17.79 ± 4.24	20.73 ± 3.16	0.73	8.490	0.001
Support utilization	8.74 ± 2.84	9.90 ± 1.92	0.43	3.107	0.002

Data were presented as mean ± standard deviation

#### Feasibility

In the large-scale clinical investigation, the response rate of the CHF-PROM was 98.94%, and the effective rate of return was 98.92%. The average completion time of CHF-PROM was 15 min. The score distribution of each item was analyzed. No major floor or ceiling effects were found. Only 0.06% of the responses to the psychological domain were missing. These findings suggest that the CHF-PROM is feasible for use in clinical practice.

## Discussion

As a chronic disorder, CHF requires special management from patients and their families, including adjustment of daily habits, liquid management, and heart rate management. Based on detailed PROs, medical professionals can provide individual instructions to patients to improve their quality of life and reduce re-hospitalization and mortality rates [36]. We established the present CHF-PROM because of the brevity of previous HF questionnaires, which were translated directly from aboard and focused little on social support and therapy status. We applied four domains (physical domain, psychological domain, social domain, and therapeutic domain) and performed large-scale survey for the healthy subjects and patients with CHF in 8 hospitals to generate this CHF-PROM, which can more fully reflect the health status of patients with CHF.

We developed the present CHF-PROM in compliance with the development principles and processes of international scales. The CHF-PROM was developed in three stages: generation of the item pool, a pilot survey to form the preliminary scale, and use of large-scale clinical trials to form the final scale. To ensure that each selected item was sensitive, representative, and independent, we adopted different statistical methods in the process of generating the scale. The average time spent performing PRO data collection was about 15 min. This is thought to have been an acceptable time for the inpatients. During this time period, the inpatients could complete the questionnaires and provide accurate responses. The time of data collection should be controlled when performing a questionnaire-based study. The timing of data collection might have influenced the responses.

The methods employed to develop related scales are still limited to CTT [37]. Our study is innovative in that IRT was applied in addition to CTT. IRT has some advantages over CTT. Using IRT, estimation of parameters is independent the number of measured subjects. It is also possible to indicate the accuracy of the test capability [38–40]. Besides statistical methods, clinical professional knowledge was also required during the process of item selection. The item “PHD18: Do you often feel nauseous?” met the requirements of statistical methods for item selection, but it did not describe a typical symptom of CHF; therefore, we deleted this item. Ten items were removed based on joint consideration of the CTT results, IRT results, and clinical knowledge. The final scale contained 57 items, 12 subdomains, and 4 domains.

We also evaluated the reliability, validity, and feasibility of the scale for 360 patients and 100 healthy subjects. The results showed that this novel scale is a reliable instrument. The CHF-PROM was generated to overcome the deficiencies in the existing HF scales. However, this study had some limitations. First, some problems exist in the personal basic information section of the scale. Economic income and consumption levels vary among different provinces and cities, making adoption of a single evaluating system of QoL inappropriate for patients with CHF. Previous studies have reported that patients’ incomes, living conditions, life events, and education levels are the main factors influencing mental health, and among them, income most strongly affects living conditions and life events. Thus, income and educational level are included in the basic information of the scale [41]. Second, some problems exist in selection of the items. We removed four items with poor sensitivity, independence, representativeness, and discrimination in the preliminary experiment. Our results suggest that every aspect should be considered in the future design of relevant scales. Finally, although our large-scale survey has indicated that CHF-PROM is a valid instrument, our samples were collected in only a limited area and are not completely representative of all patients with CHF. To further revise and improve the scale, more efforts are needed to extract larger numbers of patients from different provinces and regions and even different countries for cross-language scale adjustment to develop a CHF-PROM with wider applicability [42]. And these adjusted versions also need a validation that must be done separately from this Chinese version.

## Conclusion

In this study, we developed a CHF-PROM that showed better reliability, validity, and feasibility than previously established scales. The CHF-PROM provided the patients a greater chance to participate in treatment decisions, suggesting that PROs can be used in more clinical trials and diagnostic settings in the future. This will allow doctors to obtain more comprehensive medical information, and PROs will become an important indicator of the end-point in curative effects.

## Additional files

Additional file 1: Formation of the CHF-PROM item pool. (DOCX 20 kb)

Additional file 2: Final version of the CHF-PROM. (DOCX 31 kb)

## Abbreviations

CAID: Cronbach’s α if item deleted
CFA: Confirmatory factor analysis
CHF-PROM: Patient-reported outcome measure for chronic heart failure
CITC: Corrected item-total correlation
CTT: Classical test theory
HF: Heart failure
HRQoL: Health-related quality of life
IRT: Item response theory
PHD: Physical domain
PROs: Patient-reported outcomes
PSD: Psychological domain
SOD: Social domain
TRD: Therapeutic domain
