# Supplementary material for: CHF-PROM: validation of a patient-reported outcome measure for patients with chronic heart failure
Source: Health Qual Life Outcomes. 2018 Mar 20;16:51. doi: 10.1186/s12955-018-0874-2 (PMC5859646; doi:10.1186/s12955-018-0874-2)
Supplement: Supplementary file 1 — Formation of the CHF-PROM item pool. (DOCX 20 kb) [file 12955_2018_874_MOESM1_ESM.docx]

Additional file 1. Formation of the CHF-PROM item pool

| Item | Item |
| --- | --- |
| PHD1.Do you feel limp? | PSD12.Do you feel life is boring? |
| PHD2.Do you feel short of breath? | PSD13.Do you often check things over and over again? |
| PHD3.Is your respiratory rate increased? | PSD14.Do you often wash your hands or count over and over again? |
| PHD4.Can you lie flat to take a rest? | PSD15.Are you scared of your illness? |
| PHD5.Do you feel chest tightening? | PSD16.Are you nervous when you are alone? |
| PHD6.Do you often cough? | PSD17.Are you afraid of empty place or street? |
| PHD7.Do you cough up phlegm? | PSD18.Do you feel people are unfriendly to you? |
| PHD8.Do you cough blood? | PSD19.Do you feel people are monitoring and talking about you? |
| PHD9.Is your face pale? | PSD20. Can you trust most people? |
| PHD10.Is your skin purple? | PSD21.Do you blame others for making troubles for you? |
| PHD11.Do you feel swelling pain in the stomach? | PSD22.Do you feel that people do not judge your achievements properly? |
| PHD12.Do you have any upper right abdominal pain? | PSD23.Do you have some unnecessary ideas in your mind? |
| PHD13.Do you have ascites? | SOD1.Do your family members care about your illness? |
| PFD14.Do you pee more times in the night? | SOD2.Did your relatives, neighbors and friends ever ask about your illness? |
| PHD15.Do you have constipation? | SOD3.Do your colleagues care about your illness? |
| PHD16.Do you have any change in your dormancy? (somnolence, wakefulness or early awakening) | SOD4Did you ever receive financial support from your relatives and friends? |
| PHD17.Do you have a poor appetite? | SOD5.Did you ever receive comfort and care from your family, relatives and friends when you were in trouble? |
| PHD18.Do you often feel nauseous? | SOD6.Are you deeply involved in controlling the risk factors of heart failure? |
| PHD19.Have you lost weight recently? | SOD7.Do you talk to others voluntarily when you are in trouble? |
| PHD20.Can you take care of yourself during the daily life? | SOD8.Do you ask for help from others when you are in trouble? |
| PHD21.Can you do ordinary housework (eg. Moving the table, sweeping the floor)? | SOD9.Do you actively take part in group and organization activities ? |
| PHD22.Can you shop daily necessities by yourself? | TRD1.Can you take medicine following the doctor’s instruction? |
| PHD23.Can you walk 1500 meters at a time? | TRD2.Can you get rid of your bad habits in daily life following the doctor’s instruction? |
| PSD1.nervous and worried more easily? | TRD3.Do you regularly come back to the hospital following the doctor’s instruction? |
| PSD2.Do you feel scared for no reason? | TRD4.Do you pay more for the treatment than you have expected? |
| PSD3.Do you feel difficult to fall asleep? | TRD5.Do you feel your doctor was kind to you during the treatment? |
| PSD4.Do you feel less concentrated and forget things easily? | TRD6.Do you think the examination are necessary? |
| PSD5.Do you lose interest in the hobbies of the past? | TRD7.Is your doctor skilled and experienced? |
| PSD6.Are you worried about your illness getting worse? | TRD8.Is the treatment at this stage effective? |
| PSD7.Do you feel tired and weak? | TRD9. Have you felt better since the treatment? |
| PSD8.Do you often want to cry? | TRD10. Have you feel strong since the treatment? |
| PSD9.Do you feel depressed and not interested in anything? | TRD11.Have you felt more confident about life since the treatment? |
| PSD10.Do you feel discouraged, pessimistic and desperate about your illness? | TRD12.Are you satisfied with the medical service you have received? |
| PSD11.Can you perform your work as usual? |  |
