# Supplementary material for: CHF-PROM: validation of a patient-reported outcome measure for patients with chronic heart failure
Source: Health Qual Life Outcomes. 2018 Mar 20;16:51. doi: 10.1186/s12955-018-0874-2 (PMC5859646; doi:10.1186/s12955-018-0874-2)
Supplement: Supplementary file 2 — Final version of the CHF-PROM. (DOCX 31 kb) [file 12955_2018_874_MOESM2_ESM.docx]

Additional file 2. Final version of the CHF-PROM

Thank you for filling in this questionnaire.This is to evaluate your health conditions in the past two months to help the doctors take appropriate treatment and rehabilitation measures. Please read every item carefully,and then choose the right answer according to your actual situation in the last two weeks.Your cooperation is very important for your future treatment. We will keep your information strictly confidential. This investigation is completely voluntary. You can withdraw at any time. Thank you very much for your participation.

|  | Never | Occasionally | About half of the time | Often | Almost everyday |
| --- | --- | --- | --- | --- | --- |
| A. Physical Domain |  |  |  |  |  |
| 1. I feel short of breath. | 0 | 1 | 2 | 3 | 4 |
| 2. Is your respiratory rate increased. | 0 | 1 | 2 | 3 | 4 |
| 3. I feel chest tightening. | 0 | 1 | 2 | 3 | 4 |
| 4. I cough. | 0 | 1 | 2 | 3 | 4 |
| 5. I cough up phlegm. | 0 | 1 | 2 | 3 | 4 |
| 6. My face is pale. | 0 | 1 | 2 | 3 | 4 |
| 7. My lips are purple. | 0 | 1 | 2 | 3 | 4 |
| 8. I suffer from swelling pain in the stomach. | 0 | 1 | 2 | 3 | 4 |
| 9. I have to pee more times in the night. | 0 | 1 | 2 | 3 | 4 |
| 10. I have some changes in my dormancy. (somnolence, insomnia or early awakening) | 0 | 1 | 2 | 3 | 4 |
| 11. I have eaten less recently. | 0 | 1 | 2 | 3 | 4 |
| 12. I have lost weight recently. | 0 | 1 | 2 | 3 | 4 |
| 13. I can take care of myself during the daily life. | 0 | 1 | 2 | 3 | 4 |
| 14. I can do ordinary housework (e.g. moving the table, sweeping the floor). | 0 | 1 | 2 | 3 | 4 |
| 15. I can shop daily necessities by myself. | 0 | 1 | 2 | 3 | 4 |
| 16. I can go up to the second or third floor at a time. | 0 | 1 | 2 | 3 | 4 |
| B. Psychological Domain |  |  |  |  |  |
| 1. I feel worried easily. | 0 | 1 | 2 | 3 | 4 |
| 2. I feel scared for no reason.. | 0 | 1 | 2 | 3 | 4 |
| 3. I feel difficult to fall asleep. | 0 | 1 | 2 | 3 | 4 |
| 4. I cannot concentrate on one thing. | 0 | 1 | 2 | 3 | 4 |
| 5. My memory has been failing recently. | 0 | 1 | 2 | 3 | 4 |
| 6. I lose interest in the hobbies of the past. | 0 | 1 | 2 | 3 | 4 |
| 7. I am worried about my illness. | 0 | 1 | 2 | 3 | 4 |
| 8. I feel tired and weak. | 0 | 1 | 2 | 3 | 4 |
| 9. I often want to cry. | 0 | 1 | 2 | 3 | 4 |
| 10. I feel depressed and not interested in anything. | 0 | 1 | 2 | 3 | 4 |
| 11. I feel discouraged, pessimistic and desperate about my illness. | 0 | 1 | 2 | 3 | 4 |
| 12. I have some unnecessary ideas in my mind? | 0 | 1 | 2 | 3 | 4 |
| 13. I feel my illness is a burden to my family. | 0 | 1 | 2 | 3 | 4 |
| 14. I feel life is boring. | 0 | 1 | 2 | 3 | 4 |
| 15. I am scared of my illness. | 0 | 1 | 2 | 3 | 4 |
| 16. I feel nervous when I am alone. | 0 | 1 | 2 | 3 | 4 |
| 17. I am afraid of staying in empty place or street. | 0 | 1 | 2 | 3 | 4 |
| 18. I feel people are unfriendly to me. | 0 | 1 | 2 | 3 | 4 |
| 19. I feel people are talking about me behind my back. | 0 | 1 | 2 | 3 | 4 |
| 20. I feel few people are trustworthy. | 0 | 1 | 2 | 3 | 4 |
| 21. I blame others for making troubles for me. | 0 | 1 | 2 | 3 | 4 |
| C. Social Domain |  |  |  |  |  |
| 1. My family members care about my illness. | 0 | 1 | 2 | 3 | 4 |
| 2. My relatives, neighbors and friends have asked about my illness. | 0 | 1 | 2 | 3 | 4 |
| 3. My colleagues care about my illness. | 0 | 1 | 2 | 3 | 4 |
| 4. I have received financial support from my relatives and friends. | 0 | 1 | 2 | 3 | 4 |
| 5. I have received comfort and care from my family, relatives and friends when I was in trouble. | 0 | 1 | 2 | 3 | 4 |
| 6. I am deeply involved in controlling the risk factors of heart failure. | 0 | 1 | 2 | 3 | 4 |
| 7. I talk to others voluntarily when I am in trouble. | 0 | 1 | 2 | 3 | 4 |
| 8. I ask for help from others when I am in trouble. | 0 | 1 | 2 | 3 | 4 |
| D. Therapeutic Domain |  |  |  |  |  |
| 1. I can take medicine following the doctor’s instruction. | 0 | 1 | 2 | 3 | 4 |
| 2. I can get rid of my bad habits in daily life following the doctor’s instruction. | 0 | 1 | 2 | 3 | 4 |
| 3. I regularly come back to the hospital following the doctor’s instruction. | 0 | 1 | 2 | 3 | 4 |
| 4. I feel my doctor is kindly to me during the treatment. | 0 | 1 | 2 | 3 | 4 |
| 5. My doctor is skilled and experienced. | 0 | 1 | 2 | 3 | 4 |
| 6. Treatment at this stage was effective. | 0 | 1 | 2 | 3 | 4 |
| 7. I have felt better since the treatment. | 0 | 1 | 2 | 3 | 4 |
| 8. I have felt stronger since the treatment. | 0 | 1 | 2 | 3 | 4 |
| 9. I have felt more confident of life since the treatment. | 0 | 1 | 2 | 3 | 4 |
| 10. I am satisfied with the medical service. | 0 | 1 | 2 | 3 | 4 |
| 11. I know the side effects of drugs. | 0 | 1 | 2 | 3 | 4 |
| 12. I am worried about the side effects of drugs. | 0 | 1 | 2 | 3 | 4 |
